# Supplementary material for: Incidence of macular hole in patients undergoing pars plana vitrectomy for submacular hemorrhage
Source: PLoS One. 2025 Feb 27;20(2):e0319266. doi: 10.1371/journal.pone.0319266 (PMC11867310; doi:10.1371/journal.pone.0319266)
Supplement: Attachment — Submitted filename: Response to Reviewers2.docx [file pone.0319266.s004.docx]

Response to Reviewers letter

Dr. Jiro Kogo

Academic Editor, PLOS ONE

Dear Dr. Kogo, and Reviewers,

Manuscript ID PONE-D-24-29059

Title: Incidence of macular hole in patients undergoing pars plana vitrectomy for submacular hemorrhage

Thank you very much for your careful review that contained important suggestions to improve our manuscript. We have studied the comments carefully and have made corrections, which we hope meet your approval.

For clarity, reviewers’ comments are reproduced in Red Italics and are followed by our responses.

***Reviewer #1***

*I would like to express my gratitude to the authors for their substantial contribution to the scientific knowledge conveyed in this study. Additionally, the limitations related to the retrospective data analysis have been emphasized more clearly. I have a few minor recommendations for the authors.

Introduction and abstract and Table:
- It is advisable to use the genera term "rupture arterial macroaneurysm" in place of " rupture arterial microaneurysm."*

We corrected this term.

*Results:
− Line: 105-107
“Intravitreal t-PA injection was performed in three eyes (10.7%, day before surgery: 2, in surgery: 1) in the RAM group and two eyes (10.5%, 107 before surgery: 2) in the AMD group (p=1).”
− This statement presents challenges in interpretation. It is advisable to eliminate either surgery 1 or surgery 2 from this statement and describe using an alternative sentence.*

The sentence pointed out (Line: 105-107) was eliminated, and the following sentences were added. “In the RAM group, intravitreal t-PA injection was performed in three eyes (10.7%); the day before surgery in two eyes, and during surgery in the other eye. In the AMD group, intravitreal t-PA injection was performed in two eyes (10.5%) the day before surgery.”

*− Line 155:
“None of the patients who received intravitreal t-PA injection developed MH.”
− It would be more accurate to state the time point of injection, (preoperative, correct?).*

We have added the phrase “preoperatively or intraoperatively” to the sentence. (page9, line159)

*Table 1 and Table 2:
− Considering use:
“Preoperative intravitreal t-PA” instead of “intravitreal t-PA”.
“Intraoperative subretinal t-PA injection” instead of “subretinal t-PA injection”*
We could not make this change because “intravitreal t-PA injection” is included both intraoperatively and preoperatively.

*It is advisable to include the Mean ± SD within parentheses for continuous variables.*

In accordance with the reviewer's comment, the notation of the standard deviation has been changed.

***Reviewer #3***

*Dear Authors, it is my impression that you have put in a lot of work to revise this manuscript. However, I still have difficulties getting a clear understanding of the reporting of your research.
For example, you have stated that all the macular holes were discovered intraoperatively, this is not clearly reflected in the abstract.
Please state clearly that in all cases the MH was “discovered” during PPV after removal of the vitreous hemorrhage. This helps explain why the MH was identified only during PPV.*

We added in the abstract that all MH were found intraoperatively.

*Secondly, following your explanation in the revised letter, the incidence can not include those cases in which MH was induced by the surgical procedure or subretinal TPA injection. To give the true incidence, the sample MUST be representative of only those cases that was as a result of RAP or nAMD only and not iatrogenic. Therefore those MHs that developed following surgery have to be excluded all through the manuscript.
“Purpose: To investigate the incidence of macular hole (MH) during pars plana vitrectomy (PPV) for submacular hemorrhage (SMH) due to either retinal arterial microaneurysm (RAM) rupture or age-related macular degeneration (AMD). Methods: We retrospectively evaluated 47 eyes of 47 patients with SMH due to RAM rupture or AMD who underwent PPV. The presence or development of MHs was confirmed intraoperatively by the surgeon. We compared the incidence of MH between the RAM and AMD groups

This understanding is also lacking in the later segment below
“The MH was closed by initial surgical treatment in all eight cases. In the RAM group, subretinal tissue plasminogen activator (t-PA) injection was performed in 12 eyes. Of the 12 eyes, two developed an MH before t-PA subretinal injection. Of the remaining 10 eyes, four (40%) developed an MH intraoperatively or postoperatively”

First, please make it clear that the MH was closed during the same procedure to remove the vitreous hemorrhage. Initial surgical treatment gives the impression that a planned MH surgery was done.*

The sentence “The MH was closed by initial surgical treatment in all eight cases.” was changed to “The MH was closed by same surgical treatment that identified MH in all eight cases.” in response to the reviewer's comment (page8, line152). Abstract was also changed.

*Secondly, those eyes that developed MH after t-PA injection should be excluded for the simple reason that the intervention could be the reason for MH and not entirely the disease. So, if the injection was not done, the MH may not have occurred. This point has to be reflected in the manuscript.*

Thank you for your suggestion. You have raised an important point; however, we believe that while the incidence of MH due to the disease itself is important, another important message is that subretinal t-PA injection causes MH in RAM, but not in AMD, and therefore requires attention during surgery. We have divided the incidence of MH in the results into those due to the disease itself and those related to the surgical technique in the result (page7 , line121-124 ).We also have added the following text in the discussion to indicate the need for caution with subretinal t-PA injection, particularly in RAM rather than AMD. ” MH has not occurred in patients with AMD who have undergone subretinal t-PA injection in this study. We consider that the risk of MH may be increased when subretinal t-PA infusion is performed in patients with RAM, because patients with SMH due to RAM have a weakened fovea, even if MH did not occur preoperatively. “(page11 , line206-210)

We greatly appreciate both your help and that of the referees concerning improvements to this paper. I hope that the revised manuscript is now suitable for publication.

Sincerely yours,

Riko Matsumoto, MD, corresponding author

Department of Ophthalmology, Shiga University of Medical Science

Seta-Tsukinowacho, Otsu, Shiga 520-2192, JAPAN

Tel: +81-77-548-2276

Fax: +81-77-548-2279

e-mail: rokamoto@belle.shiga-med.ac.jp
